# Service providers’ perspectives on strategies to reduce caregiver burden among informal carers of people living with mental health conditions in rural South Africa: A qualitative descriptive study

**DOI:** 10.1371/journal.pgph.0006481

**Published:** 2026-05-20

**Authors:** Olindah Silaule, Nokuthula Gloria Nkosi, Fasloen Adams

**Affiliations:** 1 Occupational Therapy Department, University of the Witwatersrand, Johannesburg, South Africa; 2 Division of Occupational Therapy, University of Cape Town, Cape Town, South Africa; 3 Nursing Education Department, University of the Witwatersrand, Johannesburg, South Africa; 4 Division of Occupational Therapy, Stellenbosch University, Stellenbosch, South Africa; University of Sydney, AUSTRALIA

## Abstract

Caregiver burden is a public health problem affecting the emotional, physical, and mental health of informal caregivers of persons with severe mental health conditions worldwide. When caregivers feel overwhelmed, their ability to provide quality care is compromised. Although global evidence highlights the need for interventions to alleviate caregiver burden, such evidence is limited in low-resource settings, particularly in sub-Saharan Africa. This study addressed this gap by exploring service providers’ perspectives on formal and informal mental health services aimed at alleviating caregiver burden in rural South Africa. Descriptive qualitative design was adopted, and seven semi-structured individual interviews and two focus groups were conducted with twenty-nine (n = 29) mental health service providers in the Bushbuckridge municipality. Reflexive thematic analysis was performed using NVivo 12 software. Two themes were identified: caregiver and health system support strategies, and community and structural support strategies. Participants highlighted the need for caregiver-oriented mental health services and proposed multilevel strategies, including caregiver empowerment, psychoeducation, support groups, family therapy, and psychosocial rehabilitation. They also emphasised community-based support, awareness, respite and recreational services, intersectoral collaboration, and the development of targeted policies and guidelines to strengthen support for informal caregivers. The findings reflect service providers’ practical understanding of caregivers’ complex challenges and a clear vision for actionable, context-appropriate strategies to strengthen caregiver support. Given the service providers’ frontline role in mental health care, these perspectives are essential for informing the planning and implementation of interventions that are realistic, acceptable, and sustainable in low-resource settings. Future research should focus on developing and testing these strategies to enhance support systems for informal caregivers and improve outcomes for both caregivers and people living with mental health condition.

## Introduction

It is estimated that, as of 2022, approximately 1 billion people worldwide are living with a mental health condition, with more than 80% estimated to reside in low- and middle-income countries (LMICs) [1,2]. Severe mental health conditions (SMHCs), such as depression, schizophrenia, bipolar disorder, and substance use disorders, affect an estimated 4% of the global adult population and are major contributors to disability worldwide [3]. These conditions account for about 19.1% of disability-related health burdens in LMICs and represent approximately 26.1% of all reported mental health conditions in South Africa [2,4]. With a growing number of mental health conditions, mental healthcare is shifting from hospitals to families and communities [5]. Informal caregivers, including family, friends, neighbours or any member of the community providing care without remuneration, are key role players in the delivery of long-term care for persons with SMHCs [6,7]. However, studies conducted in high-income countries (HICs) and low-middle-income countries (LMICs) report that most informal caregivers experience high levels of burden in providing care to a person with a SMHC [8–10]. Caregiving has thus become a major public health issue affecting the quality of life of millions of individuals worldwide [11].

Caregiver burden is defined as the emotional, physical, and financial demands experienced by family, friends, or other individuals providing care to a person who, because of an illness or disorder, has lost the ability to carry out activities on their own [12]. As highlighted in the stress-process model of caregiving, caregiver burden is multidimensional and often determined by the interaction between caregiving demands, available resources, and the broader social and health system context [13]. This conceptual framing highlights that both individual-level stressors and system-level constraints contribute to caregivers’ vulnerability and shape the types of support they require. Studies conducted in rural South Africa reveal that informal caregivers of persons with SMHCs experienced high levels of caregiver burden attributed to the financial strain, dealing with poor self-care among care recipients, treatment non-adherence, psychotic symptoms, and disruptions in family routines, and stigmatisation from family and community [14–17]. Caregiver burden has adverse effects on caregivers’ mental, physical, and emotional health and overall quality of life, and it can also compromise recovery outcomes for persons living with SMHCs [18,19].

As frontline health workers, service providers possess unique clinical and professional expertise that is crucial for designing and implementing targeted interventions to reduce caregiver strain and strengthen family coping mechanisms [20,21]. Their system-level perspectives on the organisational and structural barriers that exacerbate caregiver burden are essential for developing interventions that are not only theoretically sound but also practical and culturally sensitive [20,22]. These insights are vital for translating policy into workable solutions that enhance treatment adherence and support long-term recovery outcomes for persons living with SMHCs [23].

To date, a range of strategies has been proposed to alleviate caregiver burden, with clinical guidelines such as those from the American Psychiatric Association and National Institute for Health and Care Excellence recommending family-based interventions that include psychoeducation and psychosocial support [24–26]. Evidence from both HICs, such as the United States of America (USA) and the United Kingdom (UK), and LMICs, including India, Brazil, Nigeria, and Botswana, shows that psychoeducation delivered individually or in groups is effective in reducing caregiver burden [27–30]. Support groups have also been identified as valuable resources for informal caregivers. In particular, mutual support groups led by caregivers or volunteers with lived experience provide safe spaces to share challenges, gain information, develop coping skills, and build confidence and psychosocial functioning [31].

While studies in both HICs and LMICs have examined strategies to alleviate caregiver burden, very few have explored the perspectives of service providers, despite their central role in organising and delivering mental health services. In South Africa, this gap is especially important given the high burden of SMHCs and the reliance on informal caregiving within resource-constrained settings [16,17]. Gaining insight into service providers’ perspectives is essential for identifying feasible, context-responsive strategies for supporting informal caregivers and strengthening community-based mental health care [17]. This study aimed to explore the formal and informal mental health services for alleviating the burden among the informal caregivers of persons with SMHCs in rural South Africa from a service provider’s perspective.

## Study methodology

### Ethics statement

The Human Research Ethics Committee of the University of the Witwatersrand (M200957) and the Mpumalanga Department of Health Research Committee (MP_202010_010) approved the study protocol. Before data collection, each participant received an information sheet outlining the purpose of the study and assurances of confidentiality and anonymity, and two separate consents were obtained: one for participation and another for audio recording of the interviews. Anonymity was ensured by assigning each participant a participant code.

### Study context

This study took place in Bushbuckridge municipality in the Ehlanzeni district in the north-eastern rural part of Mpumalanga province, South Africa. This municipality has a population size of 750,821 [32]. There is one regional hospital, two district hospitals, and 34 primary health clinics situated within the Bushbuckridge municipality. Currently, the regional hospital and one district hospital offer 72-hour observation, and one district hospital has a mental health unit with a bed capacity of 50, which is the largest in the entire Mpumalanga province. This mental health unit offers in- and outpatient services to mental health care users (MHCUs) with a variety of mental health conditions, ranging from acute to chronic and forensic cases. Therefore, all persons living with SMHCs requiring long-term and/or inpatient care are referred to this facility for further management. Once discharged into the care of their families, MHCUs are referred to the primary health clinics to collect their psychotropic medications.

### Study design and population

This study employed a qualitative descriptive design to explore the experiences of mental healthcare service providers in Bushbuckridge Municipality, South Africa [33]. The study adhered to the Consolidated Criteria for Reporting Qualitative Research (COREQ) to ensure rigour and transparency [34]. Purposive sampling was used to recruit mental healthcare service providers working at various levels of the healthcare system. Eligible participants were (i) 18 years or older; (ii) employed by the Mpumalanga Department of Health and providing services within the Bushbuckridge Municipality; and (iii) employed in their current positions for longer than six months. In total, twenty-nine (n = 29) participants (Table 1) were included in the study between August 2022 and January 2023. All targeted cadres, including mental health coordinators, mental health professionals, primary healthcare supervisors, and community health workers, were successfully recruited, and no group was under-represented in the final sample.

**Table 1:** Participant characteristics.

Identity of Participant	Format of Data Collection	Setting of Data Collection	Number
Mental health coordinators(MHC)			
Provincial	SSII	Workplace	2
District	SSII	Workplace	1
Sub district	SSII	Online	1
Mental health professionals(MHP)			
Medical officer	SSII	Workplace	1
Nursing manager	SSII	Workplace	1
Social worker	SSII	Workplace	1
Occupational Therapist	SSII	Workplace	2
Community-based service providers			
Primary healthcare supervisors (PHCS)	FG	Sub district offices	5
Community health workers (CHW)	FG	Sub district offices	15
Total			29

Abbreviations: SSII- semi-structured individual interviews; FG- focus groups.

### Data collection method and procedure

This study employed semi-structured individual interviews (SSII) and focus groups (FGs) to collect data from service providers (n = 29). Individual semi-structured interviews were conducted with mental health coordinators (MHCs) at the provincial, district, and sub-district levels, as well as with mental health professionals (MHPs) working at a district hospital in Bushbuckridge municipality. Focus groups were held with primary healthcare supervisors (PHCS) and community health workers (CHWs) based within local communities in the same municipality. These methods were chosen to capture a broad range of perspectives from mental health service providers working across various levels of the healthcare system. Focus groups, in particular, offered a cost-effective and time-efficient approach for obtaining comprehensive collective insights on strategies to support informal caregivers in rural South Africa.

The primary investigator (PI), the first author (OS), conducted semi-structured individual interviews with mental health service providers under the guidance of research supervisors (FA and NGN). The PI reached out to potential participants by phone, explained the purpose of the study, and invited them to participate. All individuals contacted agreed to participate. A suitable date, time, and location for the interviews were arranged to ensure convenience for the participants. Interviews lasted no longer than one hour and, as preferred by the participants, were held at their workplaces. This setup not only provided convenience but also allowed the researcher to gain a deeper understanding of the environment in which mental health services were delivered. In total, nine (n = 9) SSIIs were conducted. Semi-structured individual interviews were held with mental health coordinators (MHCs, n = 4) at the provincial, district, and sub-district levels, and five with mental health professionals (MHPs, n = 5) at the local district hospital, including a medical officer, nursing manager, social worker, and two occupational therapists (Table 1).

Data collection was facilitated using a demographic questionnaire and an interview guide, both developed by the PI based on existing literature, with input from research supervisors [33]. Participants were asked to share their perspectives on the formal and informal community mental health services they considered effective in reducing the burden of care experienced by informal caregivers of individuals with SMHCs. Frequency counts of codes were employed to assess data saturation, with transcripts and recordings being reviewed regularly after each interview. Data saturation was considered reached when the introduction of new codes significantly decreased.

Two FGs were conducted with primary health care supervisors (PHCS; n = 5) and community health workers (CHWs; n = 15) in the Bushbuckridge sub-district (Table 1). The FG of 15 CHWs exceeds the commonly recommended group size of 6–10 participants for optimal FG dynamics, and this was therefore not ideal [35]. However, limited transport access to the central meeting place and the homogeneity of CHWs who work closely together made conducting a single combined FG the only feasible approach to avoid excluding key perspectives. To mitigate the limitations of a larger group, participants first recorded their views individually on post-it notes and then shared them sequentially to ensure equal participation. Additionally, the inclusion of a research assistant who managed turn-taking and note-taking in line with qualitative best practice for FGs, enhanced inclusivity and data quality [35].

For convenience of participants, the two FGs were held concurrently, which required two separate facilitation teams. The first team comprised the primary investigator and a minute taker, while the second team included a skilled focus group facilitator and a minute taker. The primary investigator coordinated arrangements for the FGs in collaboration with the Bushbuckridge sub-district coordinator. The sessions were scheduled to coincide with the PHCS’s monthly meeting. The primary investigator facilitated the CHW group, and a skilled moderator facilitated the PHCS group. To ensure a consistent approach, the two facilitators met before data collection to agree on procedures to be followed during the discussions.

A semi-structured focus group guide was developed to ensure consistency in facilitation and to guide exploration of participants’ perspectives regarding the challenges of informal caregiving and the mental health services available to support informal caregivers. The guide was reviewed for content validity by four experts: two lecturers, a staff nurse, and an occupational therapist with mental health expertise. Each discussion began with an open-ended question aimed at eliciting participants’ observations of informal caregivers supporting individuals with mental disorders in their communities, followed by questions that specifically explored service providers’ perspectives on caregiver burden and the availability of formal and informal mental health services for supporting caregivers of persons living with SMHCs.

The FGs were held in separate venues within the Bushbuckridge sub-district offices and lasted approximately 1.5 hours. Data collection was terminated when no new themes or insights appeared to be emerging from the discussions, which was taken as an indication that data saturation had likely been reached. Following the sessions, the two teams debriefed, and detailed field notes were compiled to complement the interview data. Written informed consent was obtained from all participants, including permission for participation and audio recording of the sessions.

The PHCS FG and the individual interviews were conducted in English, while the CHW focus group was conducted in Xitsonga, the participants’ home language. All recordings were transcribed verbatim and translated into English by a language specialist affiliated with a local research institute in Bushbuckridge. In preparation for data analysis, the primary investigator reviewed the audio recordings while reading the transcripts, addressing any omissions or errors to ensure the accuracy of the translation.

### Data analysis

All transcripts were uploaded to NVivo 12 software for analysis. Data were analysed systematically following a six-phase, reflexive, thematic inductive approach proposed by Braun and Clarke [36], which enabled the researchers to identify meanings and patterns within the data. For data familiarisation, the PI read the transcripts continuously while concurrently listening to the recordings. Familiarisation notes were made and served as a guide for generating ideas for codes. Coding was done systematically and inductively, with each code assigned a specific description to ensure uniformity in the coding process. The first author (OS) drafted the initial codebook, drawing on the study aims and an initial open coding of subsets from transcripts of all participant groups to ensure breadth. Two co-authors (FA, NGN) then independently reviewed the draft codebook for conceptual clarity, overlap, and alignment with the research questions. Revisions focused on consolidating overlapping codes, tightening operational definitions, and adding exception codes to reduce confirmation bias. Following this, codes were grouped to form patterns and generate initial sub-themes and themes. Themes were refined and renamed through reading the coded extracts. This was done to ensure that identified codes merged coherently into a pattern to form a specific theme. The data set and familiarisation notes were read to ascertain that no codes were missed and to check the validity of the themes. Trustworthiness was ensured by inviting an experienced qualitative researcher to conduct an audit trail on the analysis process. This was further ensured by conducting peer briefings and presenting the findings to the research supervisors. To enhance the credibility of the findings, participants were given access to the transcripts for review and invited to provide feedback or add any information they considered missing. The PI practised reflexivity by keeping a journal where all thoughts and observations used to guide the analysis process were documented. In addition, the research team engaged in reflexive practice through regular peer briefings during analysis meetings, where emerging interpretations were examined, and underlying assumptions discussed [37]. Reflexive check-ins were also incorporated at each codebook update to document how team deliberations shaped code definitions and theme development [38].

### Findings

There were 29 service providers who participated in this study, including four mental health coordinators at the provincial, district, and sub-district levels; five mental health professionals from the mental health unit of a district hospital within Bushbuckridge municipality; five primary healthcare supervisors; and 15 CHWs. Their years of experience ranged from 2 to 34 years (Table 1).

The analysis of the semi-structured interviews and focus groups led to identification of the following two themes, which are set out in Table 2:

**Table 2:** Themes and subthemes of service providers’ perspectives on caregiver support.

Theme	Sub-theme
Caregiver and Health System Support Strategies	Caregiver-driven strategies
	Health worker-driven strategies
	Strengthening mental health services
Community and Structural Support Strategies	Community-based strategies
	Intersectoral strategies
	Policy and legislative strategies
The voices of the participants are reflected in this paper by using direct quotes.	

### Theme 1: Caregiver and health system support strategies

The service providers expressed their perspectives on the types of support caregivers require, including self-directed strategies, structured guidance from mental health practitioners, and strengthened clinic-based mental health services.

### Caregiver-driven strategies

The service providers identified informal caregivers as active role players in implementing strategies for alleviating their own caregiving burden. They emphasised caregiver empowerment was identified as a key strategy to strengthening caregivers’ capacity to cope with the emotional, behavioural, and practical demands associated with caring for MHCUs. This included creating opportunities for caregivers to build skills that help them accept, understand, and manage the conditions of their care recipients. As one MHC noted:

“They need to be empowered to change their circumstances”. (MHC, SSII).

Training informal caregivers to become peer facilitators of caregiver support groups was identified as a strategy to strengthen social connectedness and encourage the development of supportive networks among caregivers. Service providers acknowledge peer-led support groups as a way to enhance shared problem-solving and reduce isolation by enabling them to support one another. One MHP explained it as follows:

“The caregiver could run the support group. I heard of them where I was getting training. We observed that where there are support groups in the community, there are facilitators responsible for these support groups. Then, the health care worker can go there to give support in that particular support group”. (MHP, SSII)

### Health worker-driven strategies

The service providers identified caregiver psychoeducation as a predominant strategy for alleviating burden among the informal caregivers. They emphasised that effective psychoeducation should be practical, tailored toward a specific condition, and delivered by MHPs to improve medication management and early help-seeking. One MHP explained it as follows:

“With the psychoeducation I think it is just the basics about the mental illness, like for example, the causes… and also medication management … like explaining how the patient must take the medication and also explaining the side effects and that it is important to bring them to see the doctor …, and the symptoms that they might see like the warning signs of relapse so that the caregiver will know when to bring the user back to the hospital”. (MHP, SSII)

Service providers highlighted the importance of involving the entire family in caregiving, and as such, they identified family therapy as a strategy for strengthening support to primary caregivers. They explained that when all family members understand the mental health condition and the associated caregiving demands, the burden does not rest solely on one individual, and the household is better equipped to provide necessary support. As one PHCS explained:

“Family therapy, where not only the caregiver gets it but the rest of the family, for them to support the person who is looking after the mental healthcare user. The rest of the family needs to be involved, and family therapy will assist”. (PHCS, FG)

Facilitating the development of skills for handling MHCUs and structuring their engagement in activities was identified as a strategy for alleviating burden among the informal caregivers. Occupational therapists were identified as suitable professionals for facilitating skills training among the informal caregivers, as shown in the following comment:

“I think they need to be skilled, given information on how to manage these people in their homes. I think that is the job of the OT [occupational therapist]. I think that is the best because the patients may have time when they are home to do something with their hands, and the OTs can assist”. (MHP, SSII)

Lastly, service providers emphasised the importance of building a good rapport with the informal caregivers as a foundational strategy for creating a safe and trusting space in which caregivers feel comfortable expressing their challenges. Establishing this relationship was seen as essential for encouraging open communication, enabling caregivers to share concerns that might otherwise remain unspoken, and strengthening the continuity of support.

For example, a CHW said,

“As CHWs, we should build rapport with the caregivers so that they can be able to open up to us about their problems”. (CHW, FG)

### Strengthening mental health services

Training of MHPs and lay health workers was identified as a strategy for increasing access to community mental health services, with service providers suggesting that such training should be expanded to include caregiver-orientated components. They also highlighted the need for regular refresher training courses to ensure that mental health professions, including nurses remain updated and confident in delivering comprehensive support to MHCUs and their caregivers. This was reflected in the following comments:

“Another strategy can be training in communities like the lay health counsellors; in terms of mental health … it would help as they would assist with screening”. (MHC, SSII)

“I think if we can have the refresher courses for mental health for the nurses that could perhaps revive us”. (PHCS, FG).

CHWs expressed that training strategies should also focus on strengthening their professional identity and increasing community awareness of their role. They explained that their ability to provide services is often hindered by community members not recognising them as health workers, which affects trust and access to households. To address this, CHWs suggested that visible identifiers, such as name tags, could help legitimise their role and improve acceptance within the community. As one CHW explained:

“The clinics they are failing to give us the nametags. When we are doing fieldwork, people are chasing us away. Some people say that they do not know us because we do not have anything to identify us”. (CHW, FG)

### Theme 2: Community and structural support strategies

In this theme service providers reflected on the broader community- and system-level supports required to sustain caregiver wellbeing beyond the confines of clinical care, including community-based initiatives, intersectoral collaboration, and supportive policy and legislative strategies.

### Community-based strategies

The service providers identified community-based strategies to help alleviate the burden experienced by informal caregivers. They emphasised that support groups and counselling services should be situated within the community to improve accessibility, encourage peer support, and provide caregivers with practical coping, stress management and conflict resolution skills that enhance their ability to manage the daily challenges associated with caring for MHCUs. These perspectives were reflected in the following:

“In terms of the support groups, where it is clearly their platform for them to discuss the challenges, and I guess it is probably more about education, where they are educated on the coping skills, stress management, conflict management, or communication skills. This is just to equip them so that they can be able to deal better with the mental health care user at home”. (MHP, SSII)

The importance of leveraging existing community resources, such as community dialogues and local radio stations, to raise awareness about mental health conditions was emphasised by participants. These approaches were viewed as vital for addressing stigmatisation and building support for the informal caregivers within the community, as shown in the following comments:

“We should also have the community dialogues where community members can be invited and they can raise their myths, their understanding of the MHCU’s behaviour, and they can be educated on their behaviour and how to deal with them in a positive way”. (PHCS, FG)

Another PHCS highlighted the potential of radio programming:

“Another point I have here is the radio slot where they can discuss this matter and others can call, including those who are mentally ill, and if they are listening, they may be able to understand that they are not alone”. (PHCS, FG).

Some service providers highlighted a need for recreational facilities to give caregivers opportunities for rest, enjoyment, and temporary relief from their caregiving responsibilities. As one MHP explained:

“The recreational facilities are also needed because they may give the caregiver some relief to relax while they are out to entertain themselves” (MHP, SSII).

The service providers identified the need for community-based facilities, including day centres, sheltered employment, and respite care facilities, to better support both MHCUs and their caregivers. Day centres and sheltered employment were regarded as important for developing independent living skills among the MHCUs, thereby reducing their dependence on caregivers. In addition, respite care facilities were identified as essential for proving temporary relief when caregivers become overwhelmed. These perspectives were reflected in the following comments:

“For the mental health care users, there is this thing called the sheltered employment”. (MHC, SSII)

“What I know is that if the caregiver is no longer coping, we need to relieve them, and the institutions would serve the purpose of relieving the caregiver”. (MHP, SII)

### Intersectoral strategies

Intersectoral collaboration between health and other government sectors was identified as a strategy for alleviating informal caregiver burden. Service providers indicated that platforms such as case flow enable coordinated responses by bringing together key stakeholders to discuss and plan support for MHCUs and their families. Case flow was described as a structured forum where representatives from health, municipalities, the South African Police Services, forensics, the Department of Home Affairs, and the Department of Social Development work collectively to address challenges and strengthen community-based support systems. One MHP said:

“In this case flow, there was the Department of Home Affairs, Social Services, South African Police Services, municipality, the forensics, Justice … all the departments were coming together and discussing the cases. It was simpler and very helpful”. (MHP, SSII)

In addition, service providers highlighted the importance of intersectoral collaboration between the Departments of Health, Social Development, and Education to strengthen mental health service provision within communities. They explained that shared budgeting and coordinated planning across these sectors could increase the number of lay health workers, such as CHWs who play a crucial role in supporting MHCUs and their caregivers. This was captured in the following:

“DSD [Department of Social Development] must come with a category. Education must have its category, and when they come together …, they can make a huge category. All of them should collaborate, and if we are assisted with funds, we can appoint more CHWs”. (MHC, SSII)

Collaborations between health and community stakeholders were identified as an important way of strengthening community support for informal caregivers. Service providers emphasised that engaging religious organisations, traditional healers, municipality ward councillors, and traditional leaders could help mobilise community awareness, encourage collective responsibility, and create culturally grounded platforms for discussing caregiver needs. One PHCS explained it as follows:

“I have included the churches, traditional healers, and the community involvement by the stakeholders, such as the ward committees, the Indunas [traditional leaders] and all those that are part of the leadership in the community. They can discuss the issue of caregivers, share ideas, and discuss this with the community during their Imbizos [community gatherings]”. (PHCS, FG)

### Policy and legislative strategies

The service providers identified policy and legislative strategies as important for alleviating the burden experienced by informal caregivers. They emphasised the need for policies and guidelines that explicitly address caregiver-orientated services and strengthen the formal support available for caregivers. Participants also highlighted that such policies must be aligned with adequate financial resources to ensure implementation. These were highlighted in the following:

“What we need to have is to develop policies and put them in line with the finance but also now to presume straight to implementations so that we move forward. The policies should be in place to address this problem”. (MHC, SSII)

Service providers further indicated that policies aimed at addressing caregiver burden should be practical and easy to implement, with clearly defined action points. They emphasised that such action points should specify the required resources, confirm their availability, and identify the responsible individuals responsible for driving the implementation process. In addition, the need for a quality assurance mechanism was highlighted as a critical component in implementing policies aimed at improving access to caregiver-oriented mental health services. As one MHP explained:

“I am interested in policies if there is interest in implementing them. I am interested in knowing if there is a respite centre if there are health care workers trained, and if there is anyone who is being allocated in every ward or clinic to run a support group. The first step would be piloting with the mental champion at the clinic”. (MHP, SSII)

## Discussion

This study identified two overarching themes: caregiver and health system support strategies and community and structural support strategies. The first theme encompassed caregiver-driven and health worker-driven interventions and underscored the importance of strengthening mental health services. The second theme highlighted community-based approaches, intersectoral collaboration, and the need for policy and legislative strategies to enhance support for informal caregivers. These themes reflect caregiver burden as a multidimensional construct encompassing emotional, physical, financial, and social strain, shaped by interactions between individual caregiving demands, available support resources, and broader health system constraints within rural South Africa. As a result, addressing caregiver burden requires coordinated, multi-level strategies that operate across individual, community, and health system domains.

The service providers identified informal caregivers as active partners/participants in the implementation of strategies aimed at alleviating their burden. As such, caregiver empowerment was identified as a core caregiver-driven strategy for alleviating burden among the informal caregivers. Perlick et al. [39] identify empowerment as an effective way of reducing physical and mental burdens among family caregivers by building self-efficacy and acquiring ongoing social support networks. In rural South Africa, the prominence of empowerment strategies may also reflect systemic gaps in formal mental health services, where caregivers are required to assume expanded roles in treatment and daily care. While empowerment can promote coping and ownership of the caregiving role, an overreliance on caregiver-led strategies risks shifting responsibility from under-resourced systems onto already burdened individuals, highlighting the need for parallel system-level strengthening. Informal caregivers can nonetheless benefit from increased knowledge and skills for managing MHCUs, navigating available services, managing stress, and participating in peer-led support groups.

Mental health professionals, particularly occupational therapists, were identified as suitable for training informal caregivers in skills to manage MHCUs and structure meaningful activity engagement. This finding reflects an appreciation of functional recovery as central to both service user wellbeing and caregiver relief, given occupational therapists’ expertise in addressing limitations that affect participation in activities of daily living [40]. Through supporting activities of daily living, such as household tasks, meal preparation, managing medical appointments and medication, shopping, financial management, and personal care, occupational therapists can simultaneously reduce caregiver burden and enhance MHCUs’ independence [40,41].

Similar to previous studies [31,42], the service providers highlighted peer support groups as a strategy for building social support networks for caregivers. These groups were viewed as safe spaces for sharing experiences, developing caregiving and coping skills, and managing stress and conflict. Peer support groups primarily address the emotional and social dimensions of caregiver burden, which are often exacerbated by isolation and stigma in rural communities [42].

Although support groups are cost-effective and beneficial [31,43], challenges such as irregular attendance, dominant group members, and over-expression of distress can undermine their effectiveness. To overcome these challenges, Worrall et al. [43] recommend training professional workers, peer facilitators, and volunteer group leaders to ensure effective facilitation.

Psychoeducation emerged as a predominant health worker-driven strategy for alleviating caregiver burden. Studies conducted in Nigeria [30] and Iran [44] revealed the effectiveness of psychoeducation in reducing caregiver burden among family caregivers of persons with schizophrenia and mood disorders. Importantly, these findings suggest that psychoeducation should move beyond information provision toward skills-based approaches that directly mitigate emotional stress, improve communication with MHCUs, and enhance caregivers’ capacity to mobilise resources. Skills related to help-seeking, income-generating activities, and daily management are particularly relevant in low-resource settings where caregivers often face compounding financial pressures [45,46].

Task shifting through training community health workers (CHWs) was identified as a feasible strategy consistent with evidence from LMICs [47,48]. In South Africa, the use of CHWs to deliver basic mental health counselling has been endorsed as a viable option of expanding access beyond primary healthcare facilities [49]. In rural contexts characterised by shortages of specialist staff and geographical barriers, community-based counselling offers a pragmatic response to caregiver emotional burden. However, without adequate supervision, remuneration, and resources, task-shifting risks transferring systemic strain onto CHWs, potentially reproducing similar dynamics of burden at the community level [48]. To be effective, training should be complemented by sustained supervision and adequate resource availability [50–52].

Strengthening community support was identified as another key strategy for alleviating the burden. Service providers emphasised tangible and emotional support from family and community as protective factors. Ong et al. [53] similarly report that social support builds resilience and mediates caregiver burden. Access to community-based services such as day centres and respite care responds directly to the physical and emotional demands of caregiving by providing temporary relief and continuity of care [49,54]. Although such services are well established in high-income countries [55], their implementation in LMICs remains constrained by cost and financing challenges. Community-based rehabilitation models that leverage intersectoral collaboration offer a potentially cost-effective approach in South Africa [56,57].

Collaboration between mental health professionals and alternative care providers, including traditional healers and religious organisations, was also identified [58–59]. Such collaborations are particularly prominent in rural South Africa, where pluralistic healthcare seeking is common, and stigma remains a significant contributor to caregiver distress [60,61]. While policy support for intersectoral collaboration exists, implementation challenges persist [62,63]. Establishing clear referral pathways that recognise alternative providers as contributors to multidisciplinary care may strengthen caregiver support [64].

Finally, service providers highlighted the need for practical, caregiver-oriented policies. Beach et al. [65] emphasise that such policies should extend beyond relieving burden to building sustainable support for the caregivers to attain positive emotions, engage in activities of their choice, and build meaningful relationships. In the South African context, caregiver-oriented policies must recognise the cumulative impact of emotional strain, financial hardship, and limited social protection, and must include enforceable implementation mechanisms rather than aspirational statements alone [65].

### Implications for practice and policy

The findings demonstrate that caregiver burden in rural South Africa is shaped by intersecting individual, community, and structural factors, requiring multi-level intervention approaches. There is an urgent need for strengthening caregiver-oriented mental health services, particularly in low-resource settings where informal caregivers play a crucial role. These services can be improved by using practical approaches that are responsive to the needs of the community.

Core approaches include empowering caregivers with skills and support, equipping non-specialist health workers, such as CHWs and lay counsellors, with training and continued supervision, as well as strengthening referral pathways and continuity of care. Community-based resources like day centres and respite services should be explored using cost-effective, locally appropriate models. Intersectoral collaboration across health, social development, and community structures is essential for providing holistic support. Finally, caregiver support must be clearly integrated into policy with actionable steps, defined responsibilities, and enough resources to ensure these caregiver-support strategies are carried out effectively.

### Limitations

The study focused on service providers who were identified as offering outpatient and community-based mental health services to MHCUs and informal caregivers. As a result, the study excluded health professionals from the two hospitals that offer 72-hour mental health services and this limited the extent of the evidence gathered. Conducting interviews in the participants’ workplace was ideal; however, the interviews were constantly interrupted as some needed to be paused to allow the participants to attend to their work, which interrupted the flow and depth of information shared by the participants. The focus groups with the CHWs was larger than the commonly recommended size. Although measures were put in place to support equal participation, a group of this size may still have limited the depth of individual contributions or influenced how freely some participants engaged. Lastly, the CHW focus group was conducted in Xitsonga and subsequently translated into English by a language specialist, which may have introduced subtle shifts in meaning affecting the interpretation of the data.

## Conclusion

This study contributes important evidence on how mental health service providers in rural South Africa conceptualise feasible, context‑appropriate strategies for supporting informal caregivers of individuals living with SMHCs. Their perspectives highlight the need for strengthening caregiver‑oriented services through approaches that are practical, community‑anchored, and responsive to local resource constraints. The perspectives offered by service providers underscore the importance of task‑shifting, improved intersectoral collaboration, and the development of actionable policies that clearly define responsibilities and implementation pathways. These findings demonstrate the importance of engaging frontline workers in designing support systems that are sustainable and sensitive to the realities of caregiving in under‑resourced settings. Future work should focus on translating these insights into co‑designed interventions and evaluating their effectiveness in improving the wellbeing of caregivers and the individuals they support.
